# Supplementary material for: Universal wing- and fin-beat frequency scaling
Source: PLoS One. 2024 Jun 5;19(6):e0303834. doi: 10.1371/journal.pone.0303834 (PMC11152310; doi:10.1371/journal.pone.0303834)
Supplement: S1 File — (PDF) [file pone.0303834.s001.pdf]

Universal Wing- and Fin-beat Frequency Scaling. Supporting Information.

Jens Højgaard Jensen[1], Jeppe C. Dyre[1], Tina Hecksher[1*],

1 "Glass & Time", IMFUFA, Department of Science and Environment, Roskilde University, P.O. Box 260, DK-4000 Roskilde, Denmark

* Corresponding author

E-mail: tihe@ruc.dk (TH)

S1. LINKS TO PHOTOS OF FIG. 2

Whale

Licence: CC0

https://www.rawpixel.com/image/4022862/whale-original-public-domain-image-from-flickr

Seagulls

License CC0

https://www.pexels.com/photo/white-seagulls-flying-over-the- ocean-54462/

Hummingbird

Owner: Charles J Sharp

Licence CC BY 4.0

https://commons.wikimedia.org/wiki/File:Cinnamon_hummingbird_(Amazilia_rutila)_in_flight_Los_Tarrale

Moth

Licence: CC BY 2.0

https://www.flickr.com/photos/52450054@N04/35499910606

Mosquito

Licence: CC0

https://www.pexels.com/photo/black-white-mosquito-86722/

Bumblebee

Licence: CC0

https://www.pexels.com/photo/bumble-bee-on-yellow-daisy-67560/

Dragonfly

License: CC0

https://www.pexels.com/photo/yellow-and-black-dragonfly-on-green-stem-during-daytime-56010/

Moth

Owner: Judy Gallagher (Licence: CC BY 2.0)

https://www.flickr.com/photos/52450054@N04/35499910606

Beetle

Owner: Thorbjørn Ramløv

Private photo, reproduced with permission

Penguin

Owner: Hans Ramløv

Private photo, reproduced with permission

S2. DATA

Below all data points used in Figs. 1-3 in the main text is tabulated and referenced.

Bats

From Bullen *et al*[1].

Mass, m [kg]	Wing area, A [m^2]	Frequency, f [s^{-1}]	Symbol

0.0134	0.01788	9.04	◁
0.007	0.01351	10.91	◁
0.0065	0.01218	11.27	◁
0.0044	0.01061	10.91	◁
0.13	0.09478	6.96	◁
0.0101	0.01674	9.1	◁
0.0086	0.00959	9.34	◁
0.0071	0.01561	11.43	◁
0.0057	0.01222	10.94	◁
0.01	0.01597	10.4	◁
0.011	0.01734	10.56	◁
0.142	0.02027	11.08	◁
0.7	0.2582	3.4	◁
0.412	0.165	4.15	◁
0.0086	0.01507	9.76	◁
0.0462	0.03945	8.36	◁
0.008	0.0113	11.31	◁
0.007	0.0101	11.59	◁
0.0353	0.02584	8.19	◁
0.0281	0.02811	8	◁
0.0241	0.02736	7.47	◁
0.0056	0.01042	10.68	◁
0.0047	0.00891	10.75	◁

From Berg & Rayner (1995)[2]

Mass, m [kg]	Wing area, A [m^2]	Frequency, f [s^{-1}]	Symbol
0.0043	0.0037	13.9	◁
0.0098	0.01004	10	◁

Birds

From Pennycuick (1989)[3]

Mass, m [kg]	Wing area, A [m^2]	Frequency, f [s^{-1}]	Symbol
0.398	0.0369	9.18	∘
0.95	0.0544	8.69	∘
0.62	0.0462	9.08	∘
1.35	0.214	3.93	∘
0.39	0.117	3.61	∘
0.387	0.101	3.18	∘
1.55	0.285	2.9	∘
0.95	0.203	3.05	∘
0.325	0.106	2.74	∘
0.47	0.108	3.12	∘
0.3	0.0888	3.36	∘
0.815	0.124	4.58	∘
0.37	0.0847	4.22	∘
3.01	0.262	3.53	∘
1.24	0.173	5.07	∘
1.41	0.179	5.03	∘
1.81	0.158	5.35	∘
3.39	0.45	3.01	∘
1.47	0.408	2.24	∘
1.3	0.226	3.9	∘
0.9	0.16	4.65	∘
2.5	0.493	2.68	∘
1.92	0.419	2.55	∘
0.874	0.222	2.79	∘
0.34	0.134	3.63	∘

1.55	0.442	2.99	∘
2.08	0.327	4.53	∘
4.68	0.756	2.72	∘
0.09	0.0344	5.7	∘
1.49	0.3	3.31	∘
0.132	0.0478	9.19	∘
0.0201	0.0133	8.72	∘

From Pennycuick (1996)[4]

Mass, m [kg]	Wing area, A [m^2]	Frequency, f [s^{-1}]	Symbol
8.55	0.583	2.49	∘
3.08	0.354	2.97	∘
3.24	0.326	3.14	∘
1.23	0.167	3.93	∘
0.418	0.0773	5.61	∘
0.155	0.0469	5.42	∘
0.035	0.0215	7.65	∘
0.122	0.0197	12.3	∘
0.133	0.0221	12.3	∘
2.23	0.183	5.85	∘
1.69	0.241	3.95	∘
0.89	0.228	3.46	∘
0.61	0.105	6.35	∘
0.437	0.0646	7.62	∘
8.5	0.589	3.56	∘

From Pennycuick (2001)[5]

Mass, m [kg]	Wing area, A [m^2]	Frequency, f [s^{-1}]	Symbol

0.553	0.147	3.84	∘
0.0884	0.0251	10.6	∘
0.0228	0.013	18.2	∘
0.964	0.254	3.63	∘
0.196	0.0642	5.1	∘
0.851	0.304	2.88	∘
1.21	0.358	2.9	∘
9.01	0.682	3.38	∘
0.77	0.0829	6.83	∘
1.39	0.131	6.47	∘
2.56	0.224	4.83	∘
0.495	0.0797	5.61	∘
0.28	0.0985	3.27	∘
0.364	0.138	2.98	∘
0.925	0.2	3.13	∘
1.51	0.29	2.91	∘

From Berg & Rayner (1995)[2]. NB: in this paper wing area of one wing is given. This number is multiplied two in the table below to get the total wing area.

Mass, m [kg]	Wing area, A [m^2]	Frequency, f [s^{-1}]	Symbol
2.14	0.3994	2.64	∘
0.569	0.08452	4.9	∘
0.279	0.0754	4.48	∘
0.771	0.2284	3.19	∘
0.132	0.01836	17.8	∘
0.256	0.08844	3.5	∘
0.1	0.013	9.43	∘
0.691	0.04832	9.4	∘
0.293	0.0628	6.71	∘

0.0152	0.0103	9.35	◦
0.015	0.0093	16.1	◦
0.0562	0.02556	8.55	◦
0.0676	0.0207	10	◦
0.0199	0.01116	17.3	◦
0.0145	0.0066	27.4	◦
0.0581	0.02416	11.3	◦
0.328	0.08396	3.97	◦

From Groom (2017)[6]

Mass, m [kg]	Wing area, A [m^2]	Frequency, f [s^{-1}]	Symbol
0.00891	0.005278	25.51	◦
0.00842	0.005334	24.88	◦
0.00921	0.005976	24.58	◦
0.00617	0.003942	29.46	◦
0.00636	0.00389	28.88	◦
0.00473	0.003728	31.28	◦
0.00508	0.003508	32.3	◦
0.00517	0.003928	31.09	◦
0.00327	0.001864	47.97	◦

From Weis-Fogh (1972)[7]

Mass, m [kg]	Wing area, A [m^2]	Frequency, f [s^{-1}]	Symbol
0.0051	0.00085	35	◦

From Greenewalt (1962)[8]

Mass, m [kg]	Wing area, A [m^2]	Frequency, f [s^{-1}]	Symbol

0.0068	0.00912	25	∘
0.0069	0.00638	27	∘
0.0068	0.00778	28	∘
0.0065	0.006	22	∘
0.0023	0.00227	58	∘
0.0021	0.00227	52	∘
0.0068	0.00696	28	∘
0.0075	0.00798	19	∘
0.004	0.00356	38	∘
0.006	0.00696	28	∘
0.0071	0.00716	24	∘
0.0089	0.0098	24	∘
0.0081	0.00842	20	∘
0.0067	0.006	20	∘
0.0046	0.00444	28	∘
0.0071	0.00656	18	∘
0.0034	0.0037	30	∘
0.0037	0.00384	32	∘
0.0042	0.00384	31	∘
0.003	0.00227	41	∘
0.0028	0.001548	78	∘
0.0028	0.001548	62	∘
0.003	0.001548	51	∘
0.0125	0.00842	22	∘
0.0062	0.00638	27	∘
0.003	0.001548	58	∘
0.0067	0.00638	22	∘
0.0041	0.00462	30	∘
0.0035	0.0034	30	∘
0.0042	0.00414	25	∘

0.0041	0.00384	20	∘
0.0043	0.00462	33	∘
0.007	0.00736	27	∘
0.007	0.00512	26	∘
0.006	0.00512	32	∘
0.0073	0.01052	15	∘
0.0052	0.00678	30	∘
0.007	0.00462	21	∘
0.0031	0.00274	31	∘
0.0078	0.00798	22	∘
0.0053	0.00446	29	∘
0.0059	0.006	19	∘
0.004	0.00384	25	∘
0.0037	0.00288	40	∘
0.0038	0.00414	28	∘
0.0035	0.0037	30	∘
0.0038	0.0034	28	∘
0.0044	0.00414	30	∘
0.0038	0.00414	25	∘
0.0048	0.00414	32	∘
0.0056	0.00546	22	∘
0.0031	0.00262	30	∘
0.0023	0.001548	48	∘
0.0024	0.00174	38	∘
0.0032	0.001844	60	∘
0.0042	0.0043	30	∘
0.007	0.00736	20	∘
0.0041	0.00356	32	∘

From Viscor & Fuster (1987)[9] (and references therein)

Mass, m [kg]	Wing area, A [m^2]	Frequency, f [s^{-1}]	Symbol
2.425	0.2716	4.2	∘
0.79	0.1122	6.3	∘
0.342	0.115	5.1	∘
2.69	0.49	3	∘
2.115	0.3934	3.9	∘
1.408	0.718	2.5	∘
1.778	0.5654	2.1	∘
0.633	0.1228	5	∘
0.83	0.1328	5.1	∘
1.105	0.1856	5	∘
0.8239	0.15742	5	∘
11.602	1.3616	2.7	∘
1.578	0.202	4.3	∘
0.87	0.1358	5	∘
0.818	0.1178	4.6	∘
0.4715	0.3518	3.2	∘
0.331	0.2812	3.2	∘
0.2365	0.2592	3.1	∘
0.8895	0.52712	2.8	∘
1.105	0.5842	2.4	∘
0.159	0.0848	4.9	∘
0.813	0.257	4.3	∘
0.2085	0.1411	3.5	∘
1.89	0.2438	4.6	∘
3.361	0.2824	4.6	∘
1.304	0.1834	9	∘
0.578	0.1236	5.8	∘
0.438	0.1244	4.1	∘
0.023	0.02156	4	∘

0.0622	0.0376	5.3	○
0.211	0.1336	2.3	○
0.1038	0.0398	5.8	○
0.768	0.235	4	○
0.18	0.0914	4.1	○
0.133	0.0732	4.2	○
1.189	0.421	2.8	○
0.367	0.2298	3	○
1.915	0.5438	2.7	○
0.261	0.1706	2.8	○
0.488	0.1934	3.3	○
0.272	0.069	5.7	○
1.01	0.0848	4.5	○
0.3877	0.12244	4.3	○
0.495	0.1594	4	○
0.13	0.0715	2.5	○
0.104	0.0838	4.8	○
0.39	0.2792	2.6	○
0.0362	0.033	10	○
0.155	0.0752	2.4	○
0.0248	0.0272	18	○
0.0177	0.02524	6	○
0.0795	0.0384	5.1	○
0.5525	0.2688	3	○
1.25	0.56278	3.5	○
0.633	0.2634	4	○
0.47	0.2116	3.6	○
0.47	0.2774	2.3	○
0.253	0.133	3.9	○
0.214	0.128	3	○

0.013	0.0182	15	○
0.0915	0.052	5.6	○
0.0225	0.02356	24	○
0.0245	0.0208	20	○
0.03	0.0202	13	○

Insects

Byrne (1988)[10] (and references therein)

Mass, m [kg]	Wing area, A [m^2]	Frequency, f [s^{-1}]	Symbol
3.3e-08	1.34e-06	168.6	◇
3.5e-08	1.65e-06	180	◇
5e-08	9.6e-07	224.2	◇
1.14e-07	1.03e-06	123.4	◇
3.34e-07	2.37e-06	90.9	◇
4.11e-07	5.26e-06	104.7	◇
4.67e-07	6.63e-06	118.1	◇
7.02e-07	1.106e-05	81.1	◇
1.2e-06	2e-05	74	◇
1.5e-06	3.7e-06	480	◇
2e-06	5.8e-06	240	◇
2e-06	5.8e-06	195	◇
5.8e-06	1.5e-05	277	◇
9.9e-06	1.69e-05	262	◇
1e-05	1.96e-05	210	◇
1.2e-05	2e-05	190	◇
1.28e-05	2.3e-05	147	◇
1.93e-05	2e-05	308	◇
2e-05	4.8e-05	114	◇

2.1e-05	0.00034	25	◊
2.13e-05	2e-05	130	◊
2.13e-05	3.5e-05	174	◊
2.2e-05	3e-05	172	◊
2.3e-05	2.4e-05	160	◊
2.32e-05	4.9e-05	138	◊
3e-05	0.000175	28	◊
3e-05	0.000111	52	◊
3.3e-05	0.00015501	62	◊
3.4e-05	4e-05	190	◊
3.7e-05	0.000853	6	◊
3.71e-05	4.5e-05	186	◊
3.85e-05	4.8e-05	196	◊
4.5e-05	4.2e-05	120	◊
4.5e-05	3.6e-05	160	◊
4.6e-05	0.00048	22	◊
5.3e-05	5.78e-05	162	◊
6.1e-05	0.0003812	39.4	◊
6.4e-05	6e-05	150	◊
6.9e-05	0.000226	48	◊
7.05e-05	4.1e-05	211	◊
7.1e-05	3.5e-05	265	◊
7.3e-05	7.4e-05	144	◊
7.3e-05	7.4e-05	210	◊
7.3e-05	7.8e-05	120	◊
7.5e-05	6.8e-05	120	◊
7.8e-05	4.2e-05	250	◊
8.5e-05	5.878e-05	240	◊
8.8e-05	0.000236	58	◊
9e-05	4.4e-05	209	◊

9e-05	8.34e-05	143	♢
0.0001008	5.738e-05	197	♢
0.000101	0.000539	27	♢
0.000104	4.5e-05	220	♢
0.000107	0.0012	21	♢
0.000109	0.000116	72	♢
0.000111	8.06e-05	164	♢
0.000112	0.000317	55	♢
0.000115	4.5e-05	220	♢
0.000124	9.2e-05	120	♢
0.000127	0.00184	12	♢
0.000127	0.0003	48	♢
0.000129	8.27e-05	181	♢
0.000133	0.00032	41	♢
0.000134	0.00108	10	♢
0.000142	0.000133	80	♢
0.000144	0.00172	10.5	♢
0.000144	0.00044	48	♢
0.000159	9e-05	135	♢
0.000165	0.0008	20	♢
0.000165	0.00075	27.6	♢
0.000168	0.0002334	32.4	♢
0.000169	7.9e-05	179	♢
0.000173	0.00104	20	♢
0.000176	0.000838	28	♢
0.000178	0.0008476	24.3	♢
0.000187	9.8e-05	110	♢
0.000189	0.000262	80	♢
0.000195	0.0014	18	♢
0.0002	0.000395	21.6	♢

0.000201	0.0005	22	◇
0.000226	9e-05	128	◇
0.000233	0.00015	100	◇
0.000237	0.0008	28	◇
0.00024	0.000133	139	◇
0.000245	0.00132	20	◇
0.000248	0.00108	20	◇
0.000264	0.00015	100	◇
0.000276	0.000184	96	◇
0.000278	0.0018	10	◇
0.000281	0.001	21	◇
0.000282	0.000379	73	◇
0.000291	0.000229	78	◇
0.000298	0.0007572	71.1	◇
0.0003	0.0036	10	◇
0.000307	0.00106	21	◇
0.000308	0.00154	22.9	◇
0.000312	0.00094	42	◇
0.000318	0.0013826	19	◇
0.000345	0.0004	85	◇
0.000352	0.0012138	25.8	◇
0.000358	0.00179	23.6	◇
0.000365	0.0010735	31.4	◇
0.000382	0.001819	26.8	◇
0.000388	0.000142	130	◇
0.000388	0.000295	56.1	◇
0.000394	0.000868	17.4	◇
0.000399	0.000167	149	◇
0.000425	0.000127	170	◇
0.000428	0.0008297	19	◇

0.00043	0.0016538	21.1	◊
0.000485	0.00078	24	◊
0.000487	0.00029	41	◊
0.000495	0.000165	90	◊
0.000508	0.0016387	26.2	◊
0.00053	0.00138	38	◊
0.000537	0.00013	86	◊
0.000545	0.0013974	30	◊
0.000547	0.000203	128	◊
0.000557	0.0012	33	◊
0.000559	0.000356	48.5	◊
0.000564	0.000545	23.5	◊
0.000567	0.00026	100	◊
0.000568	0.0001805	152	◊
0.000571	0.000492	33.1	◊
0.000595	0.0013	18	◊
0.000597	0.000445	62	◊
0.000597	0.000304	104	◊
0.000605	0.005	8	◊
0.000611	0.00178	20	◊
0.000614	0.000172	130	◊
0.000638	0.000247	62.9	◊
0.000644	0.000348	87	◊
0.000665	0.001096	14.4	◊
0.000702	0.000477	48.4	◊
0.00072	0.000809	23.4	◊
0.000752	0.000764	42	◊
0.00082	0.002102	20.5	◊
0.000829	0.000443	45	◊
0.000839	0.0005564	24.9	◊

0.00088	0.000197	156	◊
0.000926	0.000388	66	◊
0.00093	0.0020217	25.5	◊
0.00094	0.000346	98	◊
0.000961	0.000402	46	◊
0.001105	0.00126	17.9	◊
0.0012	0.00228	22	◊
0.00121	0.000548	23.7	◊
0.001555	0.001044	26	◊
0.0016	0.00205	22	◊
0.0016	0.00035	125	◊
0.001618	0.001027	28	◊
0.00163	0.00097	22.9	◊
0.001699	0.0004715	41.8	◊
0.00189	0.012	8	◊
0.0026	0.0008	33	◊
0.0027	0.001072	29.5	◊
0.002809	0.00102	39.9	◊

From Darveau *et al* (2010)[11]

Mass, m [kg]	Wing area, A [m^2]	Frequency, f [s^{-1}]	Symbol
5.5e-05	2.9249e-05	234	◊
9.4e-05	4.0136e-05	223	◊
0.000159	6.7794e-05	184	◊
0.000136	5.114e-05	200	◊
0.000112	4.4048e-05	238	◊
0.00011	4.4048e-05	214	◊
8.2e-05	3.5878e-05	233	◊
0.0001	3.7198e-05	227	◊
6.7e-05	3.8754e-05	204	◊

0.000176	7.7304e-05	181	♢
8.4e-05	4.0939e-05	207	♢
0.000544	0.00018596	135	♢
0.000441	0.00015135	150	♢
0.000875	0.0002807	105	♢
0.000699	0.00031481	86	♢
6.4e-05	2.977e-05	250	♢
0.000351	0.00010594	181	♢
0.000983	0.00031392	109	♢

From Weis-Fosh (1972)[7]

Mass, m [kg]	Wing area, A [m^2]	Frequency, f [s^{-1}]	Symbol
2e-06	2.7e-06	240	♢

Ornithopter

From Valiyff *et al* (2010)[12]

Mass, m [kg]	Wing area, A [m^2]	Frequency, f [s^{-1}]	Symbol
0.32	0.098	3.51	⋆

Penguins

From Sato *et al* (2010)[13]

Mass, m [kg]	Wing area, A [m^2]	Frequency, f [s^{-1}]	Symbol
24.9	0.047	1.47	□
12	0.041	1.55	□

5.2	0.0196	2.18	□
3.8	0.0144	2.33	□
3.8	0.0156	2.56	□
3.2	0.0172	2.29	□
1.1	0.007	3.5	□

Whales

From Gough *et al* (2021)[14]

Mass, m [kg]	Fluke area, A [m^2]	Frequency, f [s^{-1}]	Symbol
20000	3.12	0.24	△
66000	4.6	0.18	△
5500	0.77	0.38	△
12000	1.97	0.24	△
40000	2.7	0.24	△
27000	3.23	0.22	△

From Fish (1998)[15]

Mass, m [kg]	Fluke area, A [m^2]	Frequency, f [s^{-1}]	Symbol
671	0.21	1.2	△
1601	0.34	1.7	△
487	0.14	2	△
225	0.11	2	△

REFERENCES

[1]R. D. Bullen and N. L. McKenzie, "Scaling bat wingbeat frequency and amplitude," J. Exp. Biol. **205**, 2615–2626 (2002).

[2]C. van den Berg and J. M. V. Rayner, "The moment of inertia of bird wings and the inertial power requirement for flapping flight," J. Exp. Biol. **198**, 1655–1664 (1995).

[3]C. J. Pennycuick, "Predicting wingbeat frequency and wavelength of birds," J. Exp. Biol. **150**, 171–185 (1989).

[4]C. J. Pennycuick, "Wingbeat frequency of birds in steady cruising flight: New data and improved predictions," J. Exp. Biol. **199**, 1613–1618 (1996).

[5]C. J. Pennycuick, "Speeds and wingbeat frequencies of migrating birds compared with calculated benchmarks," J. Exp. Biol. **204**, 3283–3294 (2001).

[6]D. J. E. Groom, M. C. B. Toledo, and K. C. Welch Jr., "Wingbeat kinematics and energetics during weightlifting in hovering hummingbirds across an elevational gradient," J. Comp. Physiol. B **187**, 165–182 (2017).

[7]T. Weis-Fogh, "Energetics of hovering flight in hummingbirds and in drosophila," J. Exp. Biol. **56**, 79–104 (1972).

[8]C. H. Greenewalt, "Dimensional relationships for flying animals," Smithsonian **144**, 1–45 (1962).

[9]G. Viscor and J. F. Fuster, "Relationships between morphological parameters in birds with different flying habits," Comp. Biochem. Physiol. **87A**, 231–249 (1987).

[10]D. N. Byrne, S. l. Buchmann, and H. G. Spangler, "Relationship between wing loading, wingbeat frequency and body mass in homopterous insects," J. Exp. Biol. **135**, 9–21 (1988).

[11]C.-A. Darveau, P. W. Hochachka, K. C. Welch, D. W. Roubik, and R. K. Suarez, "Allometric scaling of flight energetics in Panamanian orchid bees: a comparative phylogenetic approach," J. Exp. Biol. **208**, 3581–3591 (2005).

[12]A. Valiyff, J. R. Harvey, M. B. Jones, S. M. Henbest, and J. L. Palmer, "Analysis of ornithopter-wing aerodynamics," 17th Australasian Fluid Mechanics Conference (2010).

[13]K. Sato, K. Shiomi, Y. Watanabe, Y. Watanuki, A. Takahashi, and P. J. Ponganis, "Scaling of swim speed and stroke frequency in geometrically similar penguins: they swim optimally to minimize cost of transport," Proc. R. Soc. B **277**, 707–714 (2010).

[14]W. T. Gough, H. J. Smith, M. S. Savoca, M. F. Czapanskiy, F. E.

Fish, J. Potvin, K. C. Bierlich, D. E. Cade, J. Di Clemente, J. Kennedy, P. Segre, A. Stanworth, C. Weir, and J. A. Goldbogen, "Scaling of oscillatory kinematics and froude efficiency in baleen whales," Journal of Experimental Biology **224**, jeb237586 (2021), https://journals.biologists.com/jeb/article-pdf/224/13/jeb237586/2088349/jeb237586.pdf.

[15]F. E. Fish, "Comparative Kinematics and Hydrodynamics of Odontocete Cetaceans: Morphological and Ecological Correlates With Swimming Performance," Journal of Experimental Biology **201**, 2867–2877 (1998), https://journals.biologists.com/jeb/article-pdf/201/20/2867/2591943/jexbio_201_20_2867.pdf.
