# Supplementary material for: Universal wing- and fin-beat frequency scaling
Source: PLoS One. 2024 Jun 5;19(6):e0303834. doi: 10.1371/journal.pone.0303834 (PMC11152310; doi:10.1371/journal.pone.0303834)
Supplement: Attachment — Submitted filename: WingBeatFrequency_rebuttal.pdf [file pone.0303834.s002.pdf]

REVIEWER 1

The manuscript explores whether there is a universal law that explains how wing beat frequency scales with wing area and body mass in flying and swimming animals. A power law equation is proposed, and it was validated based on the experimental data provided in the literature. There are three major areas that need improvement before I recommend it for publication in PLOS.

Reply:

We thank the referee for his/her comments that made us realize that we did not make our points clear enough. We hope that we in the following (and with the changes made in the manuscript) address the concerns raised by this referee.

1) Quality of writing

- **Some sections of the manuscript reads like text book (e.g., first paragraph of the introduction, first part of the Materials and methods section)**
- **There are many speculative sentences with no justification or**
- **The description of the collated data from the literature is missing. Without more information of the data, it is difficult to judge the validity of the assumptions made (e.g., hovering assumption)**
- **Some technical terms are used very "loosely".**
- **More details should be given about how the scaling law was derived considering fluid and internal forces of the animals.**

Reply:

We have rewritten large parts of the manuscript. In particular, we have removed the "text book"-like parts and instead explain more carefully the assumptions made. Thus it is hopefully now clear that this type of derivation does not consider dimensionless

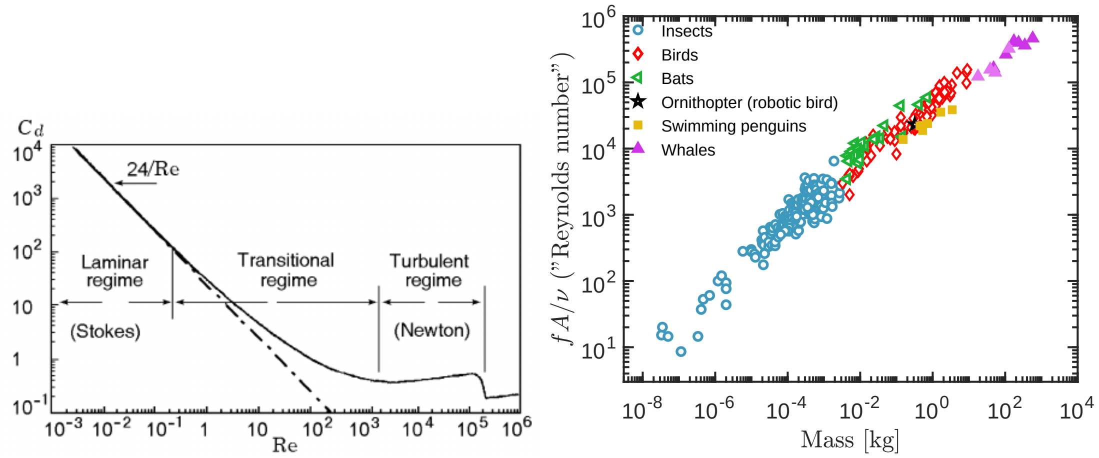

FIG. 1. Left: Drag coefficient as a function of Reynold's number. Right: Reynold's number for the data in the paper.

functions or ratios, which are all "hidden" in the dimensionless function C and could very well be different for different animals, fluid dynamic effects, or internal forces.

There are roughly speaking two regimes of fluid dynamics (see Fig. 1(left)): 1) low Reynolds numbers, where flow is laminar (drag is inversely proportional to the Reynold's number) and fluid-body interaction is dominated by momentum diffusion, which according to the Navier-Stokes equation is controlled by the viscosity η, and 2) large Reynold numbers, where fluid-body interaction is dominated by momentum transfer deriving from the body pushing the fluid aside. In the latter case, drag is roughly independent of Reynold's number. The transition happens around $\mathrm{Re} = 10$ (note that the scale in Fig. 1(left) is logarithmic). Reynolds number is defined as $\mathrm{Re} = \frac{vL}{\nu}$, where v is the streaming velocity, L is a characteristic length, and ν is the kinematic viscosity. If we take the streaming velocity to be the velocity at the tip of the wings then $v = f\,S\,\theta$, where S is the wing span, θ is the angular amplitude of the wing beat, and f is the wing-beat frequency. Taking S to be the characteristic length, we thus have $\mathrm{Re} = \frac{fS^2\theta}{\nu}$. Since we do not have θ, we plot $\frac{fA}{\nu}$ (assuming $S^2 \propto A$) as a proxy for the Reynolds number as a function of mass, see Fig. 1(right). The plot of Reynold's number versus mass (see Fig. 1(right)) shows that the Reynold's number for all data points of the paper are above 10.

We have added a paragraph to the "Results and discussion" discussing the Reynolds

number and when the wing-beat formula is expected to break down. We considered adding plot of Reynold's number to the paper, but decided against it. However, we are open to reconsidering if the reviewers find it relevant.

We stress that the derivation is made for hovering animals, not for forward flight. We do, however, *test* the formula using data for forward flight invoking the assumption that the wing-beat frequency in the two cases are not too different, which is a well-known fact reported in the literature. Therefore, there is no need to "judge the hovering assumption"; the only relevant assumption is the "same frequency" assumption. One could argue that the latter is not justified, but the fact that we find a collapse of all data points not only validates that assumption, but also reveals a near-universal constant of proportionality, which we consider a highly non-trivial fact. Admittedly, we have no good explanation for this finding, except perhaps a very general convergent-evolution-type argument.

2) Justification of the research

- **What are the research objectives, novelty compared to previous research and impact of the main findings?**

From a naive reader perspective, I don't think there is not enough context to appreciate the study.

Reply:

The wing-beat formula, $f \propto \sqrt{m}/A$ (Eq. 6), has been derived before, though with less general arguments, and this important point is now explained in more details in the manuscript. This formula must – for dimensional reasons – be correct. This means that animals with roughly similar wing shape and movement, e.g., hummingbirds or cartain classes of butterflies, should fall on a line, when their wing-beat frequency is plotted against the square-root of mass divided by wing area (Eq. 3). Different classes of animals with different wing types and wing-movement styles could give rise to different lines in the plot, reflecting different values of the prefactor (in effect, the dimensionless function C). However, the constant of proportionality varies little between different species. This

is the main – and highly non-trivial – finding of the paper: all data points fall more or less on the same line, meaning that the function C has nearly the same value for all species. Furthermore, we show that this surprising relation extends to swimming animals as well. To sum up, the novelty of this work is:

1. A proper derivation of the wing-beat formula using dimensional and physical arguments;

2. The finding that all flying animals follow this proportionality *with approximately the same prefactor*;

3. An extension of the formula to swimming animals, which are demonstrated to follow the same universal relation (when correcting for buoyancy and difference in air and water density);

4. That no physically meaningful simpler formula can describe the beat frequency of flying/swimming animals.

The immediate impact is perhaps less clear, but our aim is to report a striking and puzzling finding. It does open the question of why different classes of flying animals, despite very different wing shapes, wing movement styles, wing amplitude angles, etc., through evolution have ended up with roughly the same value of C (which is a dimensionless function of all these variables)

3) Methodology

I find simplifying the problem to "hovering behaviour" unjustified. Similarly, the assumptions of "natural frequency" of wing beats and "gravity mediated fin movements in swimming animals" (without considering wing amplitude, Reynolds number especially comparing at animals at different scales, etc.) are too simplistic and unrealistic. In addition, the evaluation of the proposed equation using r-square value is also not very informative given that the relationship between wing beat frequency, wing surface area and mass has already been established in previous studies. I think reporting results individually

one for each species is important to evaluate the generalisability of the model (including number of animals and number of n for each animal).

Reply:

We discuss the validity of the assumptions made. The main point of the paper is that *despite* these simplifications, one observes a good data collapse when plotting all species in the same plot.

Referring to the reply under 1), our main finding is the data collapse, for which reason we are puzzled by the suggestion to plot each species individually (in a way, this is already done by giving each class a different symbol and color). The R^2-value for the data collection is introduced to demonstrate that the derived wing-beat frequency relation does as good a job of describing the data as previous purely empirical power laws (Figs. 1(c+d)). References are given to each data set included in the paper. For most of these, n (number of animals) was not stated, leaving the impression that it is data for a single individual animal. For a few, e.g., the large whale data and some insect data, data points are averages over a few individuals. In principle, each individual should have its own data point in the plot since natural variations in mass and (wing/fin) area within a species should lead to slightly different flapping frequencies, but these variations are small compared to the scatter around the overall curve.

REVIEWER 2

A universal formula that relates flapping frequency to morphological parameters for propulsive locomotion through air and water is an important scientific contribution. In this paper, the authors derive Deakin's formula (that relates wingbeat frequency, body mass and wing area) by dimensional analysis and using simplifying physical assumptions. They present that the formula applies across flying animals as well as some swimming and diving animals in the context of their fin-beat frequency, once corrections in medium

density and force of buoyancy are applied. Overall, the paper is well-written, and most of the work is rigorously explained. However, the paper requires some major revisions before being published which are proposed as follows.

Reply:

We thank the referee for the thorough evaluation of the manuscript and very relevant comments.

The swimming animal data points presented in the paper are from one blue whale and a few penguins. A quick observation is that the authors do not mention the source of the blue whale data point. Nevertheless, it is an interesting observation that the data points from these swimming animals follow the same linear relationship as flying animals once the density corrections suggested by the authors are applied. However, the relationship presented for swimming animals will be impactful if it generalizes across not just penguins but also other diving animals including pinnipeds and cetaceans as well as fin-flapping fish such as batoids. Therefore, I recommend that some data from these groups of swimming animals should also be included in the paper. In case, there is a serious challenge in acquiring or presenting this data, this should be elaborated.

Reply:

We have made significant efforts to expand that part of the data collection. It is, however, more difficult because more parameters are needed. No publication that we could find reports all necessary parameters, which means that the information has to be pieced together from different sources, thus significantly increasing the uncertainty on these data points. We did find enough information to add more whale data points, but adding new categories of swimming animals (batoid fishes and pinnipeds) was not possible due to lack of sufficient data in literature. If the reviewer has suggestions for where to find more relevant data, we would be delighted to to add these – the more data, the better.

An important contribution presented in the paper is the two correction factors that account for air and water density differences. It would be useful if the authors also include the exact numerical details of how the data points from diving animals were manipulated through the correction factors before plotting them for linear regression in Fig. 2. In addition, it would also be useful to see how these data points look relative to the linear regression plot without applying the proposed correction factors. This would show the magnitude of the effect of the proposed correction factors.

Reply:

We have included more details on these corrections and now also include the uncorrected data points in Fig. 2.

Another important contribution of the paper is that the authors derive Deakin's wingbeat frequency scaling equation from "less general" arguments using conditions for Newton's third law at hovering steady-state. I think it would improve the paper's impact if the authors explain why Deakin's arguments were less general than theirs. In addition, the inclusion of a discussion of chapter 6 of "Biophysics of Insect Flight" (by N. Chari, P. Mukkavilli, L. Parayitam published by Springer) would also be useful because this chapter makes similar arguments as those used by the authors in this study.

Reply:

The manuscript now details in which regard our derivation differs from that of Deakins with the following paragraph:

> In 1970 Deakin arrived at the same result for insect flight by a dimensional analysis involving the five variables ρ_{air}, m, g, A, and f [6]. In this case, the Buckingham pi theorem of dimensional analysis [2] implies $\rho_{\mathrm{air}} A^{3/2}/m = C_1$ and $f^2 A^{1/2}/g = C_2$ where C_1 and C_2 are dimensionless, numerical constants.

To arrive at Eqs. 5 and 6 Deakin utilized the fact that it is found experimentally that $f \propto 1/A$ applies to a good approximation for insects of same mass. This means that one can write $f = \phi(m)/A$ for some function $\phi(m)$, which implies $\phi^2(m)A^{-3/2}/g = C_2$ or $A^{3/2} = \phi^2(m)/(C_2 g)$. Substituted into the C_1 Buckingham equation, this results in $\rho_{\text{air}}\phi^2(m)/(mg) = C_1 C_2$. Combining this with the experimental observation $f = \phi(m)/A$ one arrives at Eq. 5 that, as mentioned, reduces to Eq. 6 if the minor variations of air density and gravitational field strength are ignored. – The difference to the above presented physical reasoning is that the latter makes use of the fact from physics that m and g must enter into the problem in terms of the single quantity "weight", mg. This reduces the number of variables from five to four, implying that f is determined by just three input variables in which case Eq. 5 follows without empirical input.

We thank the reviewer for bringing our attention to this most relevant reference, which is now included in a discussion of other power-law expressions for the wing-beat frequency.

Nevertheless, it is interesting that despite the simplifying physical assumptions, the equation extends to non-hovering data including forward flying and diving animals. It is critical that the authors provide some physical intuition on how a relationship derived using the hovering assumption can generalize for non-hovering situations besides the argument that wing shape and movement do not have a big influence or the fact that underlying laws of physics are the same. For instance, any insights using similar steady-state conditions during hovering, forward flight and swimming can be useful.

Reply:

This is an important and difficult point, for which we have decided to best simply refer to empirical facts as reported in the literature, namely that the frequency of hovering is (fairly) close to that of regular flight. The argument for this, which we also give in the paper, is that the physiological dimensions the wings of a flying animal gives it a natural

(resonance) frequency, for which the energetic cost of the flapping movement is minimized.

Moreover, the data used or presented for wingbeat frequencies for flying animals is well-placed in the context that it is the flapping frequency close to that of hovering, or in other words, sufficient to balance the weight of the animal. Because the idea of the wingbeat frequency formula is being extended for the swimming animals, with the observation that fin-beat frequency during swimming can vary more than wingbeat frequency during flight, a natural question arises here: What value of fin-beat frequency is to be used in a comparable context? Is it the maximum value of an individual, an averaged representative value, or a value that sufficiently balances the forces on an animal underwater?

Reply:

We used an average value, whenever a range of frequencies were reported. This is now also clarified in the text.

The authors conclude that the wingbeat frequency formula with a universal proportionality constant works across species to a remarkable degree. To improve the utility of the information presented in this paper, the precise value of this proportionality constant must be given. Even though the authors give an example of the predictive capability of the formula, a detailed discussion on a wider scope of its predictive capability (for example in phylogenetic and allometric studies) would also improve the paper's impact. An important parameter in the analysis is the dimensional number C in equations 2 and 3 which incorporates the combined effects of the animal's wing shape and wing movement (excluding wingbeat frequency). In lines 128-129, the authors say that C generalizes because animals have similar wing shapes and movements. This seems to be an oversimplification because there can be large variations in wing shapes and movements even within clades, particularly insects. A more appropriate conclusion can be that the effects of wing

shapes and movements combine to have roughly similar effects across animals.

Reply:

The reviewer makes some important points here.

1) The fitted value of C is now stated in the paper. Moreover, we have rewritten the derivation to emphasize that C is a dimensionless function of dimensionless variables, which somehow for different species with different wing shapes and movement styles have evolved to give roughly the same value for C. In the paper we now state that

> This means that the data support a version of Eq. 6 with a constant of proportionality that varies little between different classes of animals. This is striking in view of the fact that the dimensionless function C in Eq. 5 could well be completely different for different animals. As the wing shapes of, e.g., a butterfly and a bat are by no means similar, it appears that evolution somehow has adjusted the dimensionless arguments going into C to give roughly the same value of C across different species.

as suggested by the reviewer.

2) The wider scope: Physics arguments lead to the wing-beat formula. This means that other functional forms (say $f \propto M^{-0.3}$) – even if giving decent correlations – cannot be derived from physical principles. This is a strong message for other phylogenetic and allometric studies. However, it must of course be kept in mind that the dimensionless function C demands a detailed analysis of each individual species (perhaps even each individual). The finding, that C is universal admittedly raises more questions than it answers. We hope zoologists will be intrigued by the data collapse and interested in explaining why nature has this preference. Unfortunately, we cannot really offer any physical intuition as to why C is a nearly universal constant (this is beyond physics), but hope that this inspires more investigation.
