# Supplementary material for: Universal wing- and fin-beat frequency scaling
Source: PLoS One. 2024 Jun 5;19(6):e0303834. doi: 10.1371/journal.pone.0303834 (PMC11152310; doi:10.1371/journal.pone.0303834)
Supplement: Attachment — Submitted filename: WingBeat_rebuttal.pdf [file pone.0303834.s003.pdf]

Rebuttal: PONE-D-23-07976R1 (Universal wing- and fin-beat frequency scaling)

Below journal requirements and reviewer comments are reproduced. Our answers and actions taken are provided in blue after each query.

Journal Requirements:

Please review your reference list to ensure that it is complete and correct. If you have cited papers that have been retracted, please include the rationale for doing so in the manuscript text, or remove these references and replace them with relevant current references. Any changes to the reference list should be mentioned in the rebuttal letter that accompanies your revised manuscript. If you need to cite a retracted article, indicate the article's retracted status in the References list and also include a citation and full reference for the retraction notice.

The reference list is complete and correct and contains no retracted papers.

Reviewers' comments:

The authors have made some significant improvements to the manuscript and addressed most of the reviewer comments. Justifications have also been provided in cases where the reviewer comments could not be fully addressed. However, I recommend following minor revisions before accepting the article.

We thank the reviewer for once again thoroughly reading our manuscript and providing relevant comments.

1. Even though the correlations look significant but please provide p values for your correlations. For instance, in Fig. 1.

A p value is the probability that the null hypothesis is correct. In most cases the null hypothesis would be that data are *not* distributed according to a Gaussian. Our dimensional analysis, however, does not make assumptions about statistical deviations, and in our opinion p values are not relevant here. The relevant measure is the Pearson correlation coefficient, which is given in the captions of Fig. 1 and Fig. 2. The paper's main figure is Fig. 2 that reports data with a Pearson correlation coefficient of 0.95. If

we blindly ask MatLab for the p value of the Fig. 1 and Fig. 2 data, vanishing numbers $\sim 10^{-200} - 10^{-130}$ are provided (a consequence of the many data points). We doubt the reader is helped by quoting these numbers and have, accordingly, not made changes to the manuscript mentioning any p values.

2. In Fig. 1 caption, I think the authors meant "blue diamonds" and "red circles".

Yes indeed. This is now changed so the caption correctly describes the plot. We are grateful this error was discovered by the reviewer.

3. Lines 69-71: It would be helpful for the reader if authors could specify whether by characterizing a wing stroke they mean kinematics, dynamics or both. Please also cite some literature when mentioning the effect of each of these parameters on the wing stroke.

We now clarify that by characterizing a wing stroke we mean in reference to kinematics. Four references have been added to the effects of the various kinematic parameters on the wing stroke frequency.

4. Line 99: The authors define the proportionality symbol here but they previously used it in line 40 and in Fig.1 caption. I'd suggest removing this line and rather replacing "simple expression" in lines 97-98 with "simple proportional relationship" or just "simple relationship" because the proportionality symbol is commonly known.

We agree and now follow the suggestion by the reviewer.

5. Before going into Results and discussion, the authors should provide algebraic details of how the correction factor is to be applied in order to account for a different medium (swimming/diving in water).

We agree with the reviewer that this does actually not belong in the "Results and discussion" section. We have moved (and slightly rewritten) the paragraph about the corrections needed to the previous section, such that all derivations and assumptions are given here.

6. Line 123-124: The authors need to more carefully phrase "wing-beat frequency is largely determined physiologically as the natural (resonance) frequency of the wing" because from recent literature we know that wingbeat frequency of many insects is supra-resonant. (For example, see Gau et al. 2022 "The hawkmoth wingbeat is not at resonance" and https://meetings.aps.org/Meeting/MAR24/Session/M38.1).

The sentence pointed out by the referee argues that the wing-beat frequency of hovering and forward flight are similar, which justifies using data from forward flight to test our wing-beat formula (derived for hovering).
The reference by the reviewer argues that the wing-beat frequency of a specific insect is higher than the resonant frequency, a conclusion that is based on comparison of measured wing-beat frequency to the resonance frequency of a mechanical model of the insect. It is reasoned that flapping at resonance may be too difficult to control. This argument must also hold for hovering and forward flight alike and thus the premise for our comparison is unchanged.
We introduced the word *largely* to reflect that the flapping frequency may not be exactly at resonance. To clarify the matter further, as suggested by the reviewer, we write (addition in italic):
"The idea is to make use of the fact that the flight wing-beat frequency is close to that of hovering [7,12]. The argument is that – like the frequency of walking – the wing-beat frequency is largely determined physiologically as the natural (resonance) frequency of the wing, where the energetic cost of the flapping movement is minimized. *The wing-beat frequency may not always be exactly at resonance for animal specific reasons (see e.g. [38]), but it will not deviate significantly (an eagle will never flap its wings at the frequency of a fly and vise versa) and, notably, whatever specific reason for a deviation from resonance must hold for hovering and forward flight alike.*"

7. The authors have put an appreciable effort into piecing together measurements from a variety of literature. I'd highly recommend that they make this data available in the supplementary material, in case they weren't planning to already. For example, in the form of a spreadsheet.

We agree. In fact, a table with all data points and appropriate references was prepared and submitted as a supplement already in the previous revision.
